# Functionalized boron nitride nanomaterials: Exploring antioxidant and antitumor activities for advanced therapeutic applications

**DOI:** 10.1016/j.isci.2026.115546

**Published:** 2026-03-30

**Authors:** Jialin Zang, Zhen Lv, Chunpeng Yang, Tianyu Zhang, Yuguang Wang, Dongxu Wang, Haiyan Liu

**Affiliations:** 1 Qiqihar Medical University, Qiqihar, Heilongjiang Province 161006, P.R. China; 2 The Second Affiliated Hospital of Qiqihar Medical University, Qiqihar Medical University, Qiqihar, Heilongjiang Province 161006, P.R. China; 3 College of Pharmacy, Qiqihar Medical University, Qiqihar, Heilongjiang Province 161006, P.R. China

**Keywords:** Chemistry, Biotechnology, Materials science

## Abstract

Fluorinated boron nitride rods (F-BNs) are functional nanomaterials with tunable surface chemistry and emerging bioapplications. Here, F-BN samples with increasing fluorination levels (F-BN-1 to F-BN-3) were synthesized, and their antioxidant activity and cellular responses were systematically evaluated. Enhanced fluorination led to progressively stronger radical scavenging, with F-BN-3 showing IC_50_ values of 31.13 ± 0.31 *μ*M (DPPH) and 26.67 ± 0.09 *μ*M (ABTS). Cellular assays revealed moderate growth inhibition by F-BN-3 in tumor cells, with IC_50_ values of 48.89 ± 0.49 *μ*M (HeLa), 27.5 ± 2.7 *μ*M (A549), and 32.9 ± 2.2 *μ*M (HepG2), while a higher IC_50_ (86.6 ± 5.4 *μ*M) in normal L02 cells indicated better tolerance. These findings demonstrate that fluorination effectively modulates BN biointeractions, enabling tunable antioxidant behavior and differential cellular responses. The low intrinsic cytotoxicity and high stability observed *in vitro* suggest promising preliminary biocompatibility and potential for biomedical applications, pending *in vivo* validation.

## Highlights

• Fluorination-level-dependent F-BN rods were constructed and characterized
• Surface fluorination enhances radical scavenging activity
• F-BN-3 selectively inhibits tumor cells with improved tolerance in normal cells
• Fluorination enables tunable biofunctional interactions and biomedical potential of BN

## Graphical abstract

**Figure:**
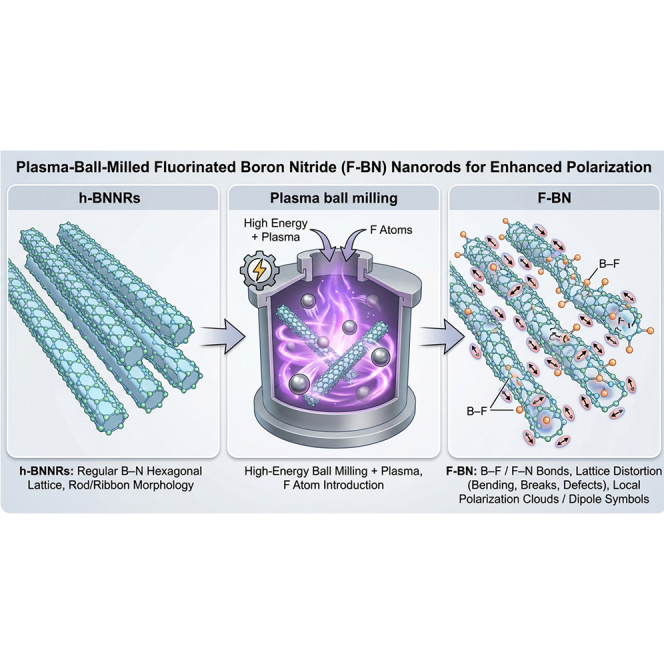

## Introduction

Boron nitride (BN) and its fluorinated derivatives have gained considerable attention in recent years due to their unique physicochemical properties, which include high thermal stability, electrical insulation, and versatile mechanical strength.^1^^,^^2^ Among the different allotropes of BN, hexagonal boron nitride (h-BN) has been widely explored for various applications in fields such as electronics, optics, and energy storage. The fluorination of BN, particularly in the form of fluorinated boron nitride nanorods (F-BNNRs), has emerged as a promising strategy to modulate its surface reactivity and enhance its biological performance. The fluorination of BN introduces polar functional groups that can significantly improve the dispersibility of BN in aqueous and organic media, making it a suitable candidate for a wide range of biomedical applications,^3^ including drug delivery,^4^ cancer therapy,^5^ and tissue engineering.^6^

The synthesis of F-BNNRs typically involves the fluorination of h-BN under controlled conditions, utilizing fluorine gas or a fluorinating agent to replace the surface-bound hydrogen atoms with fluorine.^7^ This chemical modification alters the electronic properties and surface energy of the material, which in turn affects its interaction with biological systems. Recent studies have shown that fluorinated BN nanomaterials exhibit enhanced bioactivity, including antioxidant and anticancer properties, which are critical for their potential therapeutic applications.^8^

Antioxidant activity is an essential characteristic for many biomedical materials, as reactive oxygen species (ROS) are implicated in various diseases, including cancer, neurodegeneration, and cardiovascular disorders. Antioxidants can neutralize ROS and protect cells from oxidative damage, thus offering significant therapeutic benefits.^9^^,^^10^^,^^11^ In particular, F-BNNRs have demonstrated remarkable scavenging activity against free radicals such as 2,2-diphenyl-1-picrylhydrazyl (DPPH) and 2,2′-azino-bis (3-ethylbenzothiazoline-6-sulfonic acid) (ABTS), suggesting that these materials may serve as effective antioxidants in various biological contexts.

The interaction between nanomaterials and cancer cells can lead to various cellular responses, including the inhibition of cell proliferation, induction of apoptosis, and disruption of cellular integrity.^12^^,^^13^^,^^14^ The unique combination of high surface area, chemical stability, and tunable surface chemistry of F-BNNRs allows for efficient drug loading, targeted delivery, and controlled release, making them an ideal platform for cancer therapy. Moreover, studies have shown that fluorinated BN-based nanomaterials can exhibit enhanced penetration into tumor tissues, which is critical for overcoming the limitations of conventional chemotherapy and radiation therapy.

The present study aims to investigate the preparation, antioxidant, and anticancer activities of F-BNNRs, focusing on their synthesis, characterization, and biological performance. Through systematic evaluations of antioxidant and cytotoxicity assays, this study will provide deeper insights into the potential of F-BN as multifunctional nanomaterials for biomedical applications, specifically in the fields of oxidative stress-related diseases and cancer treatment. By exploring the underlying mechanisms of these properties, this research aims to contribute to the development of novel nanomedicines and expand the utility of fluorinated BN in the growing field of nanomedicine.

## Results and discussion

### FT-IR characterization of F-BN

**Figure 1:**
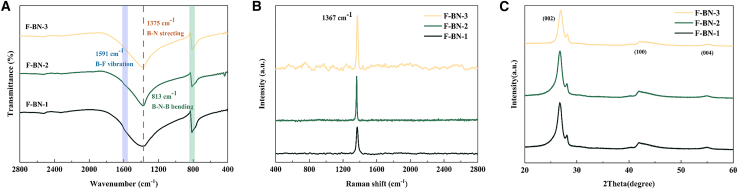
Structural characterization of F-BN (A) FT-IR spectra of F-BN-1, F-BN-2, and F-BN-3. (B) Raman spectra of F-BN-1, F-BN-2, and F-BN-3. (C) XRD patterns of F-BN-1, F-BN-2, and F-BN-3.

Figure 1A presents the FT-IR spectra of F-BN. Characteristic absorption bands are observed at 813 cm^−1^ and 1375 cm^−1^, corresponding to the out-of-plane B-N-B bending vibration and the B-N stretching vibration, respectively. In addition, a distinct absorption peak appears at 1591 cm^−1^, which is attributed to the stretching vibration of B-F bonds. Notably, the intensity of this B-F peak increases progressively with the amount of fluorinating agent employed during synthesis, indicating successful fluorination. In contrast, the N-F bond vibration is relatively weak and overlaps with the B-N-B bending mode, making it indistinguishable in the spectrum; thus, only the B-N-B peak is prominently visible in that region.^15^ Figure 1B displays the Raman spectra of F-BN, where a prominent peak centered at 1367 cm^−1^ is assigned to the characteristic B-N vibrational mode. X-ray diffraction (XRD) patterns show that all samples exhibit a prominent peak of 26.7°, 42.0°, and 55.2° corresponding to the (002), (100), and (004) crystal planes of nickel (PDF#34-0421) (Figure 1C), indicating the formation of F-BN.^16^

### XPS characterization of F-BN

**Figure 2:**
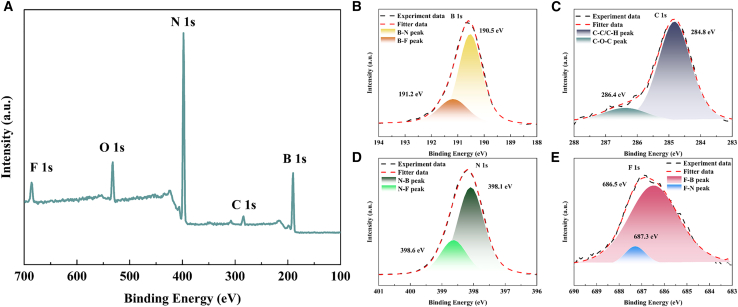
XPS analysis of F-BN-3 (A) Survey spectrum. (B) B 1s. (C) C 1s. (D) N 1s. (E) F 1s.

Figure 2 presents the XPS characterization of F-BN-3. The survey spectrum (Figure 2A) confirms the presence of B, N, F, C, and O elements, indicating successful fluorination of BN. In the high-resolution B 1s spectrum (Figure 2B), two components located at 190.5 and 191.2 eV are assigned to B-N and B-F bonds, respectively, demonstrating the incorporation of fluorine into the BN framework. The C 1s spectrum (Figure 2C) shows a dominant C-C/C=C peak at 284.8 eV with a weak C-O contribution, mainly originating from surface adsorption. As shown in Figure 2D, the N 1s spectrum exhibits a main peak at 398.1 eV corresponding to N-B bonds, accompanied by a higher-binding-energy component associated with N-F bonding. The F 1s spectrum (Figure 2E) can be deconvoluted into two peaks centered at 686.5 and 687.3 eV, attributed to F-B and F-N species, respectively. These results collectively confirm the successful fluorination of BN and the formation of multiple fluorine-related chemical environments.

### SEM and TEM characterization of F-BN

**Figure 3:**
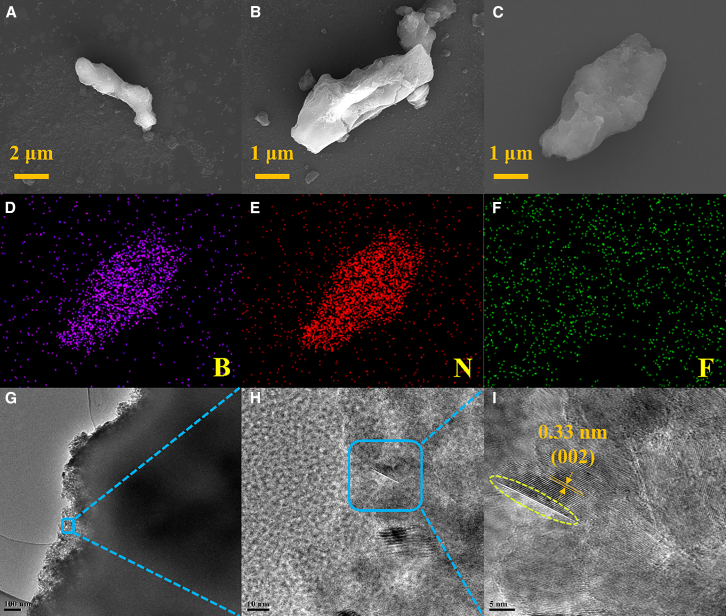
Morphological analysis of F-BN (A–C) The SEM images of F-BN-1, F-BN-2, and F-BN-3. (D–F) The elemental mapping of F-BN-1, F-BN-2, and F-BN-3. (G–I) The TEM images of F-BN-1, F-BN-2, and F-BN-3. (Scale bars, 3a: 2 *μ*m; 3b: 1 *μ*m; 3c: 1 *μ*m).

Figures 3A–3C presents the scanning electron microscopy (SEM) images of F-BN-1, F-BN-2, and F-BN-3, all of which exhibit a uniform rod-like morphology. Following fluorination, the surface of the nanorods displays a noticeable transition from a smooth to a matte appearance, indicating surface modification induced by fluorine incorporation. Elemental mapping images (Figures 3D–3F) reveal the homogeneous distribution of boron (B), nitrogen (N), and fluorine (F) across the F-BN structures, confirming the successful integration of F atoms. These observations are consistent with the XPS results, collectively validating the structural evolution of h-BNNRs upon fluorination. Figures 3G–3I show the transmission electron microscopy (TEM) images of F-BN-3 at various magnifications, further verifying the rod-like architecture observed in SEM. The high-resolution TEM (HRTEM) image of F-BN-3 (Figure 3I) reveals well-defined lattice fringes with an interplanar spacing of 0.33 nm, which corresponds to the (002) plane of h-BNNRs. Interestingly, locally expanded lattice spacings are observed on certain crystalline planes, suggesting the successful intercalation of fluorine-rich species between BN layers. This expansion is likely attributable to effective fluorine doping during the fluorination process, which disrupts the original interlayer stacking of h-BNNRs.^17^

### Contact angle measurement

**Figure 4:**
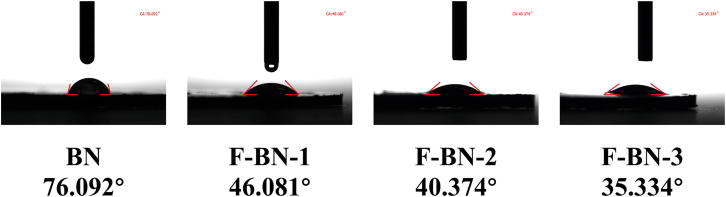
Static water contact angle images of pristine BN and F-BN samples

The surface wettability evolution of BN and fluorinated BN samples was evaluated by static water contact angle measurements, as shown in Figure 4. BN exhibits a relatively high contact angle of 76.09°, indicating its intrinsically hydrophobic surface nature. After fluorination, a pronounced decrease in contact angle is observed for all F-BN samples, demonstrating a clear enhancement in surface wettability. Specifically, the contact angles decrease to 46.08°, 40.37°, and 35.33° for F-BN-1, F-BN-2, and F-BN-3, respectively. This progressive reduction in contact angle suggests that fluorination effectively modulates the surface physicochemical properties of BN. The introduction of fluorine atoms induces localized electronic redistribution and surface polarization, which increases surface energy and promotes stronger interactions with polar water molecules. In addition, fluorination-related structural defects and surface functional groups may further facilitate water adsorption, thereby enhancing hydrophilicity. Notably, the gradual decrease in contact angle with increasing fluorination degree indicates a tunable wettability behavior, consistent with the fluorine-induced modification of surface chemistry. The improved hydrophilicity is expected to be beneficial for subsequent biological interactions, as enhanced surface wettability is often associated with improved interfacial compatibility and mass transport in aqueous environments.

### Zeta potential measurement

**Figure 5:**
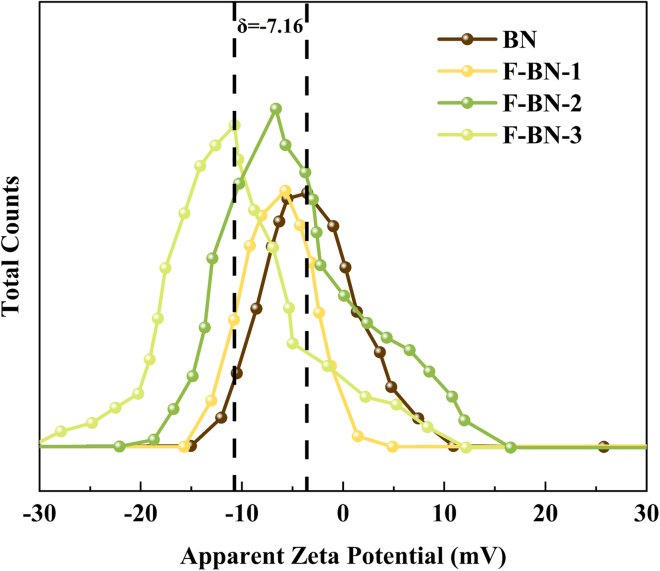
Zeta potential distributions of BN and F-BN samples

The surface charge characteristics and dispersion stability of BN and F-BN samples were investigated by zeta potential measurements, as shown in Figure 5. BN exhibits a relatively low negative zeta potential of −3.55 mV, indicating weak electrostatic repulsion between particles and a limited colloidal stability in aqueous media. After fluorination, all F-BN samples display increasingly negative zeta potential values, suggesting a progressive modification of surface charge properties. Specifically, the zeta potentials of F-BN-1, F-BN-2, and F-BN-3 are measured to be −5.70 mV, −6.64 mV, and −10.71 mV, respectively. This gradual shift toward more negative values indicates that fluorine incorporation effectively alters the surface electronic environment of BN, likely due to the introduction of electronegative F species and associated surface polarization effects. Although the absolute zeta potential values remain in a moderate range, the observed trend clearly demonstrates enhanced electrostatic repulsion and improved dispersion stability upon fluorination. In particular, F-BN-3 exhibits the most negative zeta potential, implying the strongest resistance to particle aggregation among the samples. Such improvement in suspension stability is beneficial for maintaining homogeneous dispersion in aqueous environments, which is advantageous for subsequent interfacial interactions and biological evaluations. It is worth noting that the moderate magnitude of the zeta potential is reasonable considering the intrinsically non-ionic nature of the BN framework and the surface-confined fluorination strategy employed in this work. Nevertheless, the systematic increase in negative surface charge provides clear evidence that fluorination plays an important role in tuning the colloidal behavior of BN-based materials.

The water dispersibility of BN and F-BN was evaluated by contact angle and zeta potential measurements. Fluorination leads to a clear decrease in contact angle and a progressive increase in negative surface charge, indicating enhanced surface hydrophilicity and improved suspension stability in aqueous media. Although the materials are not intrinsically soluble, their improved dispersion behavior is sufficient for biological testing and interfacial interactions.

### Antioxidant activity assay

**Figure 6:**
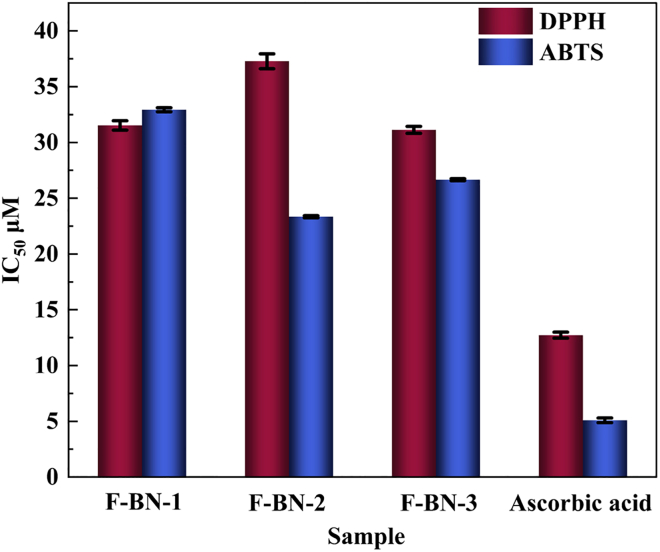
Comparison of IC_50_ values for DPPH and ABTS assays among F-BN-1, F-BN-2, and F-BN-3 (Data are represented as mean ± SEM).

Figures 6A–6C present a systematic evaluation of the antioxidant performance of three F-BN samples (F-BN-1, F-BN-2, and F-BN-3) using DPPH and ABTS radical scavenging assays, with ascorbic acid employed as a benchmark antioxidant. Overall, all samples exhibit a clear concentration-dependent radical scavenging behavior, while distinct performance differences among the fluorinated BN samples are also evident, indicating a strong dependence on fluorine incorporation and the resulting surface electronic structure.

As shown in Figure 6A, the DPPH assay reveals that increasing sample concentration leads to progressively enhanced radical inhibition for all materials. Ascorbic acid exhibits the strongest scavenging capability, approaching nearly 100% inhibition at 100 *μ*M. Among the F-BN samples, F-BN-3 displays the highest DPPH scavenging efficiency, significantly outperforming F-BN-1 and F-BN-2 and approaching the activity of ascorbic acid. This enhanced performance suggests that F-BN-3 possesses a more favorable surface electronic environment for radical interaction, which is closely related to its fluorine content and fluorine-induced defect structure. The ABTS assay results shown in Figure 6B follow a similar overall trend, although the scavenging efficiencies are generally lower, particularly at low concentrations (<10 *μ*M), indicating that ABTS radicals are more difficult to neutralize than DPPH radicals. Despite this increased difficulty, F-BN-3 consistently exhibits superior scavenging activity among the F-BN samples. This behavior implies that F-BN-3 has a higher electron-donating capability or a greater density of electronically active surface sites, which facilitates charge transfer or radical stabilization during the scavenging process.

From a mechanistic perspective, the enhanced radical scavenging performance of F-BN-3 can be attributed to the fluorine-induced modulation of the BN surface electronic structure. The formation of B-F and F-N bonds introduces a strong electronegativity contrast and localized charge redistribution, generating polarized defect sites and electron-deficient boron centers. These sites act as effective interaction centers for radical species, enabling scavenging through electron transfer, hydrogen abstraction, or radical stabilization pathways. In contrast, insufficient fluorination (as in F-BN-1) may lead to a limited density of active sites, while non-optimal fluorine distribution or excessive lattice perturbation (as suggested for F-BN-2) can reduce effective charge transfer and weaken radical capture efficiency.

Figure 6C summarizes the IC_50_ values for all samples in both radical systems, further supporting these observations. Ascorbic acid exhibits the lowest IC_50_ values, approximately 13 *μ*M for DPPH and 6 *μ*M for ABTS, confirming its superior antioxidant activity. Among the F-BN samples, F-BN-3 shows the smallest IC_50_ values (approximately 31 *μ*M for DPPH and 27 *μ*M for ABTS), outperforming F-BN-1 and F-BN-2 and demonstrating its overall optimal antioxidant performance. Notably, F-BN-2 presents the highest IC_50_ value in the DPPH assay (∼37 *μ*M), indicating less efficient radical scavenging, which may arise from unfavorable fluorine distribution, reduced surface polarization, or partial passivation of reactive sites.

### Cytotoxic activity assay

Collectively, these results demonstrate that radical scavenging activity in F-BN is not solely governed by material concentration, but is strongly correlated with fluorine-content-dependent surface electronic structure. An appropriate fluorination level, as realized in F-BN-3, achieves an optimal balance between defect density and electronic polarization, thereby maximizing radical scavenging efficiency. This tunable antioxidant behavior highlights the potential of F-BN as a functional material for biomedical applications, antioxidant coatings, and radical inhibition systems.

**Figure 7:**
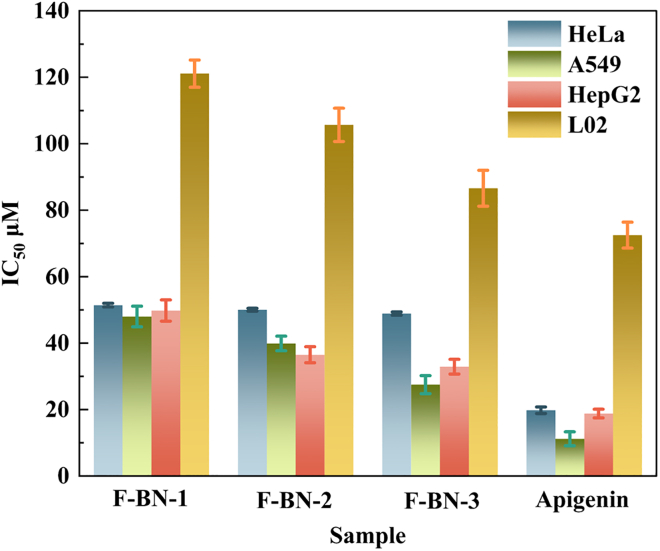
Comparative cytotoxicity of F-BN samples and apigenin against HeLa, A549, HepG2, and L02 cells (Data are represented as mean ± SEM).

Combining the results in Table 2 and Figure 7, it can be observed that the F-BN series exhibits distinguishable cell proliferation inhibitory behaviors across different cell models, with the extent of inhibition varying with both material composition and cell type. In HeLa cells, the IC_50_ values of F-BN-1, F-BN-2, and F-BN-3 are 51.43 ± 0.54 *μ*M, 50.06 ± 0.44 *μ*M, and 48.89 ± 0.49 *μ*M, respectively, indicating a slight but consistent decrease with compositional modulation. A more pronounced trend is observed in A549 cells, where the IC_50_ values decrease from 48.0 ± 3.1 *μ*M to 39.9 ± 2.2 *μ*M and further to 27.5 ± 2.7 *μ*M. A similar tendency is also evident in HepG2 cells, with corresponding IC_50_ values of 49.8 ± 3.2 *μ*M, 36.5 ± 2.4 *μ*M, and 32.9 ± 2.2 *μ*M. In contrast, the IC_50_ values of the three F-BN materials toward normal L02 cells are markedly higher, reaching 121.1 ± 4.1 *μ*M, 105.7 ± 5.0 *μ*M, and 86.6 ± 5.4 *μ*M, respectively, with relatively small standard deviations, indicating good experimental reproducibility. As a reference, Apigenin exhibits IC_50_ values of 19.80 ± 0.98 *μ*M for HeLa cells, 11.2 ± 2.1 *μ*M for A549 cells, 18.8 ± 1.3 *μ*M for HepG2 cells, and 72.5 ± 3.9 *μ*M for L02 cells. Although the overall inhibitory effect of the F-BN series is weaker than that of Apigenin, the materials display a consistent and tunable suppression trend across multiple tumor cell lines while maintaining a relatively high tolerance threshold in normal liver cells. These observations suggest that the biological response of the F-BN materials is more likely associated with mild interfacial regulation and microenvironmental perturbation rather than direct cytotoxicity. These findings suggest that the biological response of the F-BN series is not primarily governed by direct cytotoxicity, but rather by physicochemical perturbations at the material-cell interface. Fluorination introduces defect sites and polar B-F bonds, which alter surface charge distribution, local electron density, and interfacial hydration behavior. Such modifications may facilitate redox-related interfacial reactions, potentially influencing extracellular ROS balance and membrane-associated signaling events. Instead of inducing acute oxidative damage, the F-BN materials are more likely to impose a mild but sustained redox imbalance. Tumor cells, which typically maintain a higher basal ROS level and operate near their oxidative stress threshold, are intrinsically more vulnerable to further redox perturbation. Even subtle shifts in redox homeostasis may disrupt metabolic flux, mitochondrial function, or proliferative signaling pathways, thereby amplifying growth-inhibitory responses. In contrast, normal hepatocytes, with more robust antioxidant buffering capacity and lower basal oxidative stress, exhibit higher tolerance to such interfacial modulation. Therefore, the observed selective inhibition pattern can be rationalized as a redox-mediated differential sensitivity rather than nonspecific toxicity. This mechanism-oriented interpretation strengthens the potential of fluorination-induced surface engineering as a strategy for tunable bio-interactive BN materials.

**Table 2:** IC_50_ values (*μ*M) of F-BN-1, F-BN-2, and F-BN-3 against HeLa, A549, HepG2, and L02 cells

	HeLa	A549	HepG2	L02
F-BN-1	51.43 ± 0.54	48.0 ± 3.1	49.8 ± 3.2	121.1 ± 4.1
F-BN-2	50.06 ± 0.44	39.9 ± 2.2	36.5 ± 2.4	105.7 ± 5.0
F-BN-3	48.89 ± 0.49	27.5 ± 2.7	32.9 ± 2.2	86.6 ± 5.4
Apigenin	19.80 ± 0.98	11.2 ± 2.1	18.8 ± 1.3	72.5 ± 3.9

HeLa: human cervical cancer cells; A549: human lung cancer cells; HepG2: human hepatocellular carcinoma cells; L02: normal human liver cells.

Data represent the mean values of three independent determinations.

It is worth noting that previous studies have reported intrinsic cytotoxic or growth-inhibitory effects of BN-based materials, such as BN nanosheets, BN nanotubes, and BN quantum dots. However, these effects are often associated with nanoscale size effects or additional functional components. In contrast, the present work demonstrates that surface fluorination alone can effectively modulate the biological behavior of BN by tuning its surface chemistry and interfacial properties. Compared with previously reported BN systems, the fluorinated BN developed here exhibits more controllable redox-related behavior and improved dispersion stability, highlighting the advantage of fluorine-induced surface engineering.

In conclusion, the results of this study establish a clear relationship between the degree of fluorination and the bio-related responses of BN nanosheets. Among the investigated samples, F-BN-3 exhibits the most pronounced radical scavenging behavior, with IC_50_ values of 31.13 ± 0.31 *μ*M in the DPPH assay and 26.67 ± 0.09 *μ*M in the ABTS assay. In cellular evaluations, F-BN-3 induces measurable growth-inhibitory responses in multiple tumor cell lines, with IC_50_ values of 48.89 ± 0.49 *μ*M for HeLa cells, 27.5 ± 2.7 *μ*M for A549 cells, and 32.9 ± 2.2 *μ*M for HepG2 cells, while exhibiting a comparatively higher IC_50_ of 86.6 ± 5.4 *μ*M in normal L02 liver cells (Table 1). These results indicate that fluorination effectively tailors the surface chemical environment of BN, thereby modulating its interactions with reactive species and cellular systems. Rather than implying a dominant cytotoxic mechanism, the observed cellular responses are more reasonably attributed to fluorination-induced interfacial regulation and microenvironmental modulation. Collectively, this work demonstrates that controlled fluorination provides a viable materials-level strategy for engineering the biofunctional behavior of BN, offering a foundation for its further exploration in multifunctional and bio-interfaced material systems. Beyond its *in vitro* performance, the observed biofunctional properties of F-BN-3 suggest its potential as a candidate for future translational exploration in biomedical contexts, including postoperative oxidative stress modulation and tumor recurrence management. It should be noted that these proposed applications are based on cell-based evaluations and require further validation in appropriate *in vivo* models. Moreover, the generally low cytotoxicity and high physicochemical stability reported for BN-based materials indicate favorable preliminary biocompatibility, supporting the continued investigation of F-BN-3 as a proof-of-concept platform for clinically relevant therapeutic systems.

**Table 1:** Screening results for DPPH and ABTS radical scavenging activity (IC_50_*μ*M) of F-BN-1, F-BN-2, and F-BN-3

Sample	IC_50_*μ*M^a^	
	DPPH	ABTS
F-BN-1	31.53 ± 0.43	32.93 ± 0.18
F-BN-2	37.28 ± 0.67	23.34 ± 0.10
F-BN-3	31.13 ± 0.31	26.67 ± 0.09
Ascorbic acid	12.72 ± 0.274	5.0925 ± 0.209

IC_50_: half maximal inhibitory concentration; DPPH: 2,2-diphenyl-1-picrylhydrazyl; ABTS: 2,2′-azino-bis (3-ethylbenzothiazoline-6-sulfonic acid).

a Data represent the mean values of three independent determinations.

### Limitations of the study

This work provides preliminary insight into the antioxidant and cellular responses of fluorinated BN materials; however, several limitations should be noted. The biological evaluation was confined to *in vitro* assays, and the *in vivo* pharmacokinetics, biodistribution, clearance pathways, and long-term biosafety of F-BNs remain unclear. The mechanistic basis underlying fluorination-dependent bioactivity was inferred mainly from phenomenological observations rather than direct molecular evidence, and key pathways such as oxidative stress regulation and intracellular trafficking were not fully elucidated. In addition, only a limited panel of cell lines and fluorination levels was investigated, which may not capture the full spectrum of structure-activity relationships. Future studies integrating systematic surface chemistry control, mechanistic bioassays, and *in vivo* validation are necessary to establish the translational potential of F-BN nanomaterials.

## Resource availability

### Lead contact

Further information and requests for resources, materials, and data should be directed to and will be fulfilled by the lead contact: Dr. Zhen Lv (Email: pangmax27@163.com).

### Materials availability

This study did not generate new unique materials, cell lines, or biological resources. All synthesized samples (F-BN composites) are available from the lead contact upon reasonable request.

### Data and code availability

• All data reported in this paper will be shared by the lead contact upon reasonable request.
• This paper does not report any original code.
• No publicly archived datasets were generated during this study.

## Acknowledgments

This work was financially supported by the Basic Scientific Research Operating Expenses Program for Provincial Undergraduate Universities of the 10.13039/501100003851Heilongjiang Provincial Department of Education (2023-KYYWF-0885, 2022-KYYWF-0819) and the Joint Guidance Project of the Qiqihar Science and Technology Plan (LSFGG-2022038). We also appreciate the support from the Leading Talent Team in the Pharmacy of Qiqihar.

## Author contributions

J.Z.: writing – original draft, conceptualization, investigation, supervision, funding acquisition. Z.L.: Writing-review and editing, conceptualization, supervision. C.Y.: investigation. T.Z.: investigation. Y.W.: investigation. D.W.: investigation. H.L.: investigation. All authors have read and approved the final manuscript.

## Declaration of interests

The authors declare that they have no known competing financial interests or personal relationships that could have appeared to influence the work reported in this paper.

## STAR★Methods

### Key resources table

**Table:** 

REAGENT or RESOURCE	SOURCE	IDENTIFIER
**Chemicals, peptides, and recombinant proteins**		

Boric acid	Anhui Zesheng Technology Co., Ltd. (Shanghai, China)	CAS: 10043-35-3
Melamine	Anhui Zesheng Technology Co., Ltd. (Shanghai, China)	CAS: 108-78-1
N,N′-dimethylformamide (DMF)	Anhui Zesheng Technology Co., Ltd. (Shanghai, China)	CAS: 68-12-2
Dimethyl sulfoxide (DMSO)	Anhui Zesheng Technology Co., Ltd. (Shanghai, China)	CAS: 67-68-5
2,2-Di(4-tert-octylphenyl)-1-picrylhydrazyl (DPPH)	Anhui Zesheng Technology Co., Ltd. (Shanghai, China)	CAS: 84077-81-6
2,2′-azino-bis (3-ethylbenzothiazoline-6-sulfonic acid) (ABTS)	Anhui Zesheng Technology Co., Ltd. (Shanghai, China)	CAS: 30931-67-0
Nafion	Macklin Biochemical Co., Ltd. (Shanghai, China)	CAS: 31175-20-9

**Experimental models: Cell lines**		

HeLa	ATCC	Cat. No. HG-HD005
A549	ATCC	Cat No. CL-0016
HepG2	ATCC	Cat No. HB-8065
L02	ATCC	Cat No. HL7702

**Software and algorithms**		

Origin 2021	OriginLab	https://www.originlab.com
MDI Jade 9	Materials Data	https://www.materialsdata.com/prodjd.html
OMNIC	Thermo Fisher Scientific	https://www.thermofisher.cn/cn/zh
Avantage	Thermo Fisher Scientific	https://www.thermofisher.cn/cn/zh
SPSS Statistics	IBM	https://www.ibm.com/products/spssstatistics

### Method details

#### Synthesis of h-BNNRs

In a representative synthesis protocol for hexagonal boron nitride nanoribbons (h-BNNRs), boric acid (40 mmol) was first dissolved in 20 mL of deionized (DI) water under continuous magnetic stirring at 45 °C. Separately, melamine (5 mmol) was dispersed in 30 mL of DI water and stirred at 75 °C until complete dissolution. The melamine solution was then added dropwise to the boric acid solution under constant stirring, and the resulting mixture was maintained at 45 °C for an additional 6 h to promote homogeneous precursor formation. The resulting white precipitates were collected via centrifugation, washed thoroughly with DI water, and subsequently dried under vacuum at 60 °C for 12 h. The dried precursors were then subjected to thermal annealing at 1100 °C for 2 h under a nitrogen atmosphere, yielding the final h-BNNR powder.^18^

#### Synthesis of F-BN

In a typical synthesis, 0.1 g h-BNNRs and 0.1 g Nafion were dissolved in 14 mL DMF, 2 mL of ethanol and 2 mL of DI water, and transferred to a beaker for sonication for 30 min. Then, the mixture transfer to a 20 mL Teflon-lined at 200 °C for 12 h. After naturally cooling to room temperature, the resulting precipitates were isolated by high-speed centrifugation, followed by repeated washing with DI water and ethanol to remove residual impurities. The purified products were then dried in a vacuum oven at 80 °C for 48 h to afford white solid powders, denoted as F-BN-1. In addition, by varying the amount of Nafion to 0.2 g and 0.3 g under otherwise identical conditions, the corresponding products were labeled as F-BN-2 and F-BN-3, respectively.

#### Antioxidant activity

The antioxidant capacities of F-BN-1, F-BN-2 and F-BN-3 were assessed using a DPPH radical scavenging assay by monitoring the decrease in absorbance at 516 nm, corresponding to the maximum absorption of DPPH in ethanol. Owing to the limited solubility of F-BN-1, F-BN-2 and F-BN-3 in ethanol, stock solutions were first prepared in dimethyl sulfoxide (DMSO). For each assay, 1 mL of the DMSO stock solution was mixed with 9 mL of an ethanolic DPPH solution (final concentrations: DPPH, 80 *μ*M; test compound, 1 *μ*M), yielding a 10 mL reaction mixture. The samples were incubated in the dark for 30 min to prevent photodegradation. As a control, a DPPH-only solution (80 *μ*M in ethanol) was subjected to the same conditions. Absorbance was subsequently measured at 516 nm using ethanol as the blank.

The ABTS radical scavenging activity of F-BN-1, F-BN-2 and F-BN-3 were evaluated by measuring the decrease in absorbance at 734 nm, corresponding to the characteristic maximum of the ABTS⋅^+^ chromophore in ethanol.

$$\text{SR}\%=\left(\frac{control\;absorbance-sample\;absorbance\;}{control\;absorbance}\right)*100\%$$

Ascorbic acid was used as the standard antioxidant for comparison. Data were represented as means ± standard deviations of triplicate experiments.

#### Cytotoxic activity

The cytotoxic potential of F-BN-1, F-BN-2 and F-BN-3 against tumor cell lines was assessed using a standard MTT viability assay. Briefly, tumor cells were seeded into 96-well plates at a density of 5 × 10^4^ cells/well in appropriate culture medium and incubated for 24 h at 37 °C in a humidified atmosphere with 5% CO_2_. Subsequently, test compounds were added in triplicate across eight concentrations (0, 1, 2.5, 5, 10, 20, 50, and 100 *μ*M). Following a 44 h incubation period, 20 *μ*L of MTT solution (5 mg/mL in PBS) was added to each well and incubated for an additional 4 h to allow for formazan crystal formation. The supernatant was then carefully removed, and 150 *μ*L of DMSO was added to each well to solubilize the formazan crystals. In a separate confirmation step, the culture medium was replaced with 100 *μ*L of phenol red-free RPMI-1640 medium, followed by the addition of 10 *μ*L of 12 mM MTT stock solution. After 4 h of incubation at 37 °C, 85 *μ*L of medium was aspirated, and 50 *μ*L of DMSO was added to each well. The contents were mixed thoroughly and incubated for 10 min at 37 °C. Absorbance was measured at 590 nm using a microplate reader (SpectraMax iD5, Molecular Devices, USA). Cell viability (%) was calculated using the formula:

Viability (%) = (*OD*_*t*_ / *OD*_*c*_) × 100,

where *OD*_*t*_ and *OD*_*c*_ represent the mean absorbance values of treated and untreated (control) cells, respectively.

#### Characterizations

Fourier transform infrared spectroscopy (FT-IR, Nicolet 380, Thermo Scientific, USA) was employed to identify the characteristic vibrational modes of F-BN. Crystal structure and phase purity were analyzed by X-ray diffraction (XRD, D8 Advance, Bruker, Germany). Raman spectra were acquired with a confocal Raman spectrometer (DXRxi, Thermo Scientific, USA) using a 532 nm laser source. Morphological and structural features were examined using high-resolution transmission electron microscopy (HRTEM, JEM-2100F, JEOL, Japan) and field-emission scanning electron microscopy (FE-SEM, MIRA LMS, TESCAN, Czech Republic). Elemental distribution was further confirmed by energy-dispersive X-ray spectroscopy (EDS, Smartedx, Oxford Instruments, UK). X-ray photoelectron spectroscopy (XPS) was performed on a Thermo Scientific ESCALAB 250Xi spectrometer (USA) equipped with a monochromatic Al K*α* X-ray source (h*ν*=1486.6 eV) under ultrahigh vacuum (base pressure<1×10^-9^ mbar). Graphs in the main text and Supporting Information were generated from the raw data using Origin 2021. XRD patterns were analyzed using MDI Jade 9. FTIR spectra were processed using OMNIC. XPS data were analyzed using Thermo Scientific Avantage.

#### Statistical analysis

All statistical evaluations were completed via SPSS Statistics. Unless otherwise indicated, *in vitro* experiments were performed in triplicate, with data summarized as mean ± standard deviation (SD). Pairwise group comparisons relied on a two-tailed Student’s t-test, whereas multi-group comparisons were analyzed using one-way ANOVA coupled with Tukey’s post hoc test. Statistical significance was established at p < 0.05 (∗), p < 0.01 (∗∗), p < 0.001 (∗∗∗) and p > 0.05 (not significant, ns).
